# Supplementary material for: Construction and validation of a novel cuproptosis-related long noncoding RNA signature for predicting the outcome of prostate cancer
Source: Front Genet. 2022 Dec 6;13:976850. doi: 10.3389/fgene.2022.976850 (PMC9763621; doi:10.3389/fgene.2022.976850)
Supplement: Supplementary file 2 [file DataSheet1.ZIP › Source data for review purpose only/Source data/25.pRRophetic/Cor.TAE684.pdf]

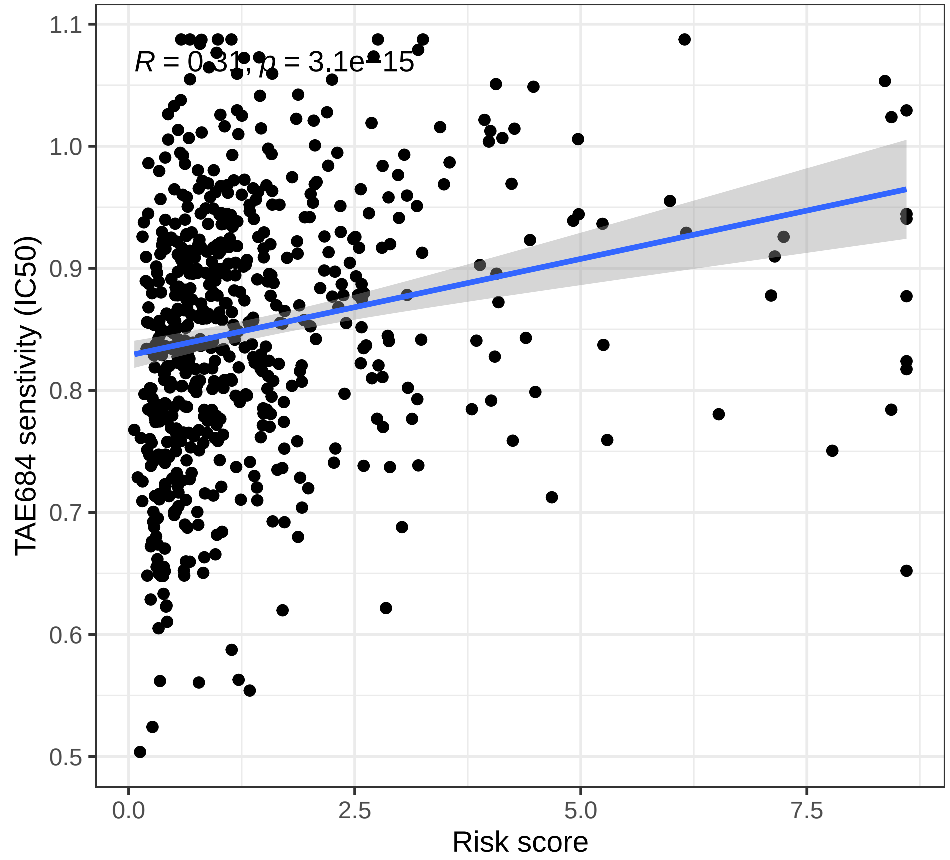

R = 0.31, p = 3.1e−15
TAE684 senstivity (IC50)
1.1
1.0
0.9
0.8
0.7
0.6
0.5
0.0
2.5
5.0
7.5
Risk score
